# Associations of electronic health literacy with related factors in individuals diagnosed with colorectal cancer: a cross-sectional analysis

**DOI:** 10.3389/fpubh.2026.1681416

**Published:** 2026-02-04

**Authors:** Min Wang, Yan-hua Xu, Zhen-qing Ren, Bei-li Hu, Yan Zhang

**Affiliations:** 1 Department of General Surgery, The Affiliated Taizhou People’s Hospital of Nanjing Medical University, Taizhou, China; 2 Department of Nursing, The Affiliated Taizhou People’s Hospital of Nanjing Medical University, Taizhou, China

**Keywords:** colorectal cancer, disease management, electronic health literacy, quality of life, self-efficacy

## Abstract

**Objective:**

This study aims to assess the current level of electronic health literacy (eHL) among individuals diagnosed with colorectal cancer (CRC) and to identify associated factors that may inform the development of targeted interventions to support patient-centered health management.

**Methods:**

A cross-sectional study was conducted involving 258 individuals with CRC, recruited using a convenience sampling method from a Class A tertiary hospital in Jiangsu Province, China. Data were collected using a general information questionnaire, the Chinese version of the eHealth Literacy Scale (eHEALS), the Multidimensional Scale of Perceived Social Support (MSPSS), the General Self-Efficacy Scale (GSES), and the European Quality of Life Five-Dimensions Questionnaire (EQ-5D). Univariate analyses and multiple linear regression were conducted to explore factors associated with eHL.

**Results:**

The mean eHEALS score was 19.71 ± 8.97, indicating a generally low level of eHL among participants. Pearson correlation analysis demonstrated significant positive correlations between eHL and scores on the GSES, MSPSS, and EQ-5D (*p* < 0.01). Multiple stepwise linear regression identified higher educational attainment, unmarried status, cohabitation with friends, presence of two or more chronic comorbidities, GSES scores, and EQ-5D scores as significant predictors of eHL (*R*^2^ = 0.658, *F* = 59.78, *p* < 0.05).

**Conclusion:**

eHL remains suboptimal among individuals with CRC in China. Interventions should focus on populations at elevated risk of low eHL, with an emphasis on enhancing self-efficacy and improving health-related quality of life to facilitate more effective engagement with electronic health resources and improve clinical outcomes.

## 1 Introduction

Colorectal cancer (CRC) constitutes a major global public health concern, with a steadily increasing disease burden worldwide (1). According to data from the Global Cancer Observatory 2022, approximately 1.926 million new cases are diagnosed annually, accompanied by nearly one million deaths. CRC ranks as the fourth most commonly diagnosed malignancy and the third leading cause of cancer-related mortality globally (2). In China, epidemiological data from 2022 report 517,000 newly diagnosed cases, positioning CRC as the fourth most prevalent malignant tumor (3). Although advancements in early detection, targeted therapies, and integrated treatment approaches have significantly improved survival outcomes, individuals diagnosed with CRC continue to face complex therapeutic regimens, frequent monitoring, and the necessity for sustained lifestyle adjustments (4). These demands highlight the need for effective health information processing and self-management capabilities.

The digital transformation of healthcare has rendered internet-based technologies indispensable platforms for health information dissemination, reshaping traditional doctor-patient communication models and fostering innovative approaches to health education. In China, the national “Internet Medical Services” initiative, coupled with the widespread adoption of virtual healthcare platforms, has deeply integrated healthcare providers and patients into a context-specific digital health ecosystem under the framework of China’s health system. Within this unique context, electronic health literacy (eHL) (5)—defined as the ability to seek, locate, comprehend, and critically appraise health-related information from electronic sources to manage or address health issues (6)—has emerged as a pivotal associated factor of health behaviors among Chinese populations. This competency is closely linked to effective engagement with digital medical resources, thereby influencing the formation of health behaviors and ultimately affecting clinical outcomes (7). However, critical gaps remain in understanding the role of eHL in Chinese colorectal cancer (CRC) patients within this digital health landscape: existing research on Chinese CRC patients is mostly descriptive, lacks theoretical framework guidance, and fails to clarify the interplay between eHL and key influencing factors (predisposing, enabling, need factors) or their joint impacts on health outcomes. Additionally, the challenges posed by the variable quality of online CRC-related health information and limited eHL in this cohort remain insufficiently explored (8).

Patients with CRC face a dual challenge: they have heightened health-information needs due to complex care pathways, yet they also encounter significant barriers in accessing and evaluating electronic information, a situation exacerbated by their disease and treatment burden. Unlike traditional health literacy, which focuses on understanding basic health information such as medical advice and printed materials, eHL emphasizes the ability to search, evaluate, and apply health information through digital technologies, representing an extension of health literacy in the digital era (9, 10). Consequently, integrating strategies to enhance eHL within clinical practice has become increasingly imperative. While factors such as age, education, and social support are known influence factors of eHL in broad populations, a nuanced understanding of these factors specifically among Chinese CRC patients is lacking (11). This gap is particularly relevant given China’s rapid rollout of “Internet Medical Services” and the unique socio-cultural context of its healthcare system. Moreover, existing research has largely centred on basic information-seeking skills; a deeper, theory-informed investigation of patients’ critical-evaluation abilities and the interplay among eHL, psychosocial factors and health outcomes within this defined clinical setting is therefore warranted (12).

Internationally, existing research on eHL in cancer/CRC populations has confirmed that eHL is a critical associated factor of health outcomes, with demographic and psychological factors identified as potential correlates. In China, however, research on eHL in CRC patients remains scarce: existing relevant studies are mostly limited to descriptive analyses of eHL levels in general cancer populations or small-scale CRC cohorts, with insufficient exploration of the mechanisms underlying eHL’s role in health management and a lack of theoretical framework-guided investigations (13). This hinders the development of targeted eHL interventions for Chinese CRC patients. To address this gap and provide a robust theoretical foundation, this study is grounded in Andersen’s Behavioral Model of Health Services Use.

This study aims to examine the association between predisposing factors (sociodemographic variables, self-efficacy measured by GSES) and enabling resources (social support measured by MSPSS, eHL measured by eHEALS) among Chinese CRC patients, explore the relationship between eHL (as an enabling resource) and need factors (health-related quality of life measured by EQ-5D) in this cohort, and identify the mediating role of eHL in the relationship between predisposing/enabling factors and health-related quality of life; the novelty of this study lies in its targeted exploration of eHL-related mechanisms in Chinese CRC patients under the guidance of Andersen’s model, filling the gap of theoretical framework deficiency in existing domestic research and generating contextually tailored insights for optimizing eHL interventions and Internet Medical Services strategies for this specific population.

### 1.1 Research question and hypotheses

*Overarching research question*: Based on Andersen’s Behavioral Model of Health Services Use, what are the relationships among predisposing factors, enabling resources (including eHL), and need factors (health-related quality of life) in Chinese CRC patients, and does eHL mediate the association between predisposing/enabling factors and health-related quality of life?

*Hypothesis*: Higher levels of predisposing factors and enabling resources are positively associated with eHL, which in turn is positively associated with better health-related quality of life (measured by EQ-5D) in Chinese CRC patients, with eHL mediating the relationships between predisposing/enabling factors and health-related quality of life after adjusting for other confounding variables.

## 2 Participants and methods

### 2.1 Theoretical framework and variable selection

This study is grounded in Andersen’s Behavioral Model of Health Services Use, which posits that health service utilization and health outcomes are determined by three interconnected categories of factors: predisposing factors, enabling resources, and need factors. This framework guides the selection of variables and the exploration of their relationships in this study, with explicit mapping between each construct of the model and the study variables as follows:

Predisposing factors refer to individual characteristics that exist prior to the occurrence of health problems and influence the willingness to engage in health behaviors, and in this study, they include sociodemographic variables (gender, age, educational level, marital status, employment status, type of medical insurance, individual income, place of residence, living situation) and self-efficacy (measured by the General Self-Efficacy Scale, GSES);

Enabling resources represent external resources that facilitate or hinder the utilization of health services and engagement in health behaviors, with corresponding variables in this study including social support (measured by the Multidimensional Scale of Perceived Social Support, MSPSS) and eHL (measured by the eHealth Literacy Scale, eHEALS) where eHL is identified as a core enabling resource linking other factors to health outcomes;

Need factors relate to individuals’ perceived or actual health needs that directly drive health service utilization and health outcome changes, and the need factor in this study is health-related quality of life measured by the European Quality of Life 5-Dimensions (EQ-5D-3L).

### 2.2 Study participants

This study was conducted in a single Class A tertiary hospital in Taizhou, Jiangsu Province, with participants recruited from the intestinal surgery ward and stoma nursing outpatient clinic of the hospital. Participants were individuals diagnosed with CRC who received care at this hospital between April 2024 and March 2025, enrolled using a convenience sampling method. The inclusion criteria were as follows: (1) a diagnosis consistent with the Chinese Protocols for Diagnosis and Treatment of Colorectal Cancer (2023 Edition) (14); (2) age ≥ 18 years; (3) the ability to communicate effectively and comprehend the questionnaire content; (4) prior experience in accessing or reading health-related information online; (5) provision of informed consent and voluntary participation. The exclusion criteria included: (1) the presence of severe cognitive impairment or diagnosed psychiatric disorders; (2) a critical clinical condition or serious complications that would impair participation.

In accordance with the requirements for multivariate analysis, Ethical approval for this study was granted by the hospital’s ethics committee (approval number: KY2024-058-01). The required sample size was calculated using the formula N = 4U*α*^2^S^2^/*δ*^2^. With α set at 0.05, Uα was 1.96, and the allowable error δ was defined as 0.25 × S. Based on previously reported data where *S* = 9.07 (15), the initial sample size was estimated to be 246. Accounting for an anticipated attrition rate of approximately 10%, the final required sample size was adjusted to 271 participants.

### 2.3 Data collection instruments

All measures were selected based on two core criteria: strict alignment with the constructs of Andersen’s Behavioral Model of Health Services Use (to ensure theoretical validity) and sufficient psychometric evidence (reliability and validity) in Chinese populations or chronic disease cohorts (to ensure cultural adaptability and measurement accuracy). Detailed justification for each instrument is provided below:

General Information Questionnaire (also referred to as Predisposing Factors Questionnaire, self-developed): This self-developed questionnaire, which functions as the general information questionnaire in this study, is explicitly designed to operationalize the “predisposing factors” construct of Andersen’s model—specifically capturing individual characteristics that pre-exist health problems and influence the willingness to engage in health behaviors. The drafting of questionnaire items followed a rigorous three-step process: first, a comprehensive review of domestic and international literature on CRC patient management, Andersen’s model application studies, and existing scales measuring predisposing factors in chronic disease populations was conducted to identify core items that are theoretically associated with predisposing factors. Second, three experts in oncology nursing and health services research were invited to conduct expert consultations; they evaluated the relevance, clarity, and comprehensiveness of each draft item, providing revisions for ambiguous expressions and suggesting supplements for missing key variables. Third, a pilot test was conducted among 20 Chinese CRC patients to assess the questionnaire’s readability, completion time, and acceptability; items with unclear understanding were revised to match the local economic conditions and cognitive level of the target population. The final version of the general information questionnaire demonstrated good content validity (content validity index, CVI = 0.94) and clarity, making it a valid tool for assessing predisposing factors in this Chinese CRC cohort.

eHealth Literacy Scale (eHEALS): This scale was selected to measure the core “enabling resource” of eHL, which is theoretically consistent with the study’s focus on digital health engagement in Chinese CRC patients. The eHEALS is the most widely used instrument for assessing eHL globally, with dimensions (finding, evaluating, and applying electronic health information) that perfectly align with the study’s definition of eHL derived from Andersen’s model. Critically, its Chinese version has been validated in multiple Chinese chronic disease populations, showing excellent internal consistency (Cronbach’s *α* = 0.931) and construct validity (confirmatory factor analysis supporting a single-factor structure, comparative fit index = 0.96) (16). Its psychometric robustness in Chinese chronic disease groups ensures accurate measurement of eHL in CRC patients, who share similar long-term disease management needs with other chronic disease populations.

Multidimensional Scale of Perceived Social Support (MSPSS): This scale was chosen to measure the “enabling resource” of social support, as its multidimensional structure (family, friends, significant others) is theoretically congruent with Andersen’s model, which emphasizes comprehensive external resources facilitating health behavior. The Chinese version of MSPSS, translated and revised by Jiang Qianjin, has been extensively validated in Chinese chronic disease cohorts, including cancer patients. Psychometric evidence shows high internal consistency (Cronbach’s *α* = 0.82 for total score and subscales) (17) and criterion validity (significantly correlated with psychological well-being, *r* = 0.45–0.52) (18). Its cultural adaptability to Chinese contexts and proven reliability in similar populations make it an optimal choice for this study.

General Self-Efficacy Scale (GSES): Selected to operationalize the “predisposing factor” of self-efficacy, this scale is theoretically aligned with Andersen’s model, which identifies inherent self-beliefs as key predisposing characteristics influencing health behavior willingness. The GSES has been widely validated in Chinese populations, particularly in chronic disease patients (including cancer), with consistent psychometric performance: internal consistency (Cronbach’s *α* = 0.922) (19) and construct validity (significantly correlated with coping styles, *r* = 0.38–0.46) (20). Its brevity (10 items) and focus on general coping self-efficacy are well-suited for CRC patients, who may face fatigue or cognitive burden from treatment, ensuring high response quality and compliance.

European Quality of Life 5-Dimensions (EQ-5D-3L): This instrument was selected to measure the “need factor” of health-related quality of life (HRQoL), as its theoretical focus on physical, psychological, and social functioning aligns with Andersen’s model’s definition of health needs driving health outcomes (21). The Chinese version of EQ-5D-3L, with a utility scoring model adapted for Chinese populations by Liu et al. (15), has been validated in Chinese CRC patients specifically, demonstrating good test–retest reliability (intraclass correlation coefficient = 0.860) and construct validity (significantly correlated with disease stage, *r* = −0.32). Each item is scored on a 3-point scale: 1 (no problems), 2 (some or moderate problems), and 3 (extreme problems). A health utility index (HUI) was calculated using the following formula: HUI = 1 − constant term − five-dimensional tax rate coefficient − N3. Resulting score range from −0.149 to 1, with higher scores reflecting better health-related quality of life (15, 22).

### 2.4 Survey methods

Participants were consecutively recruited from the intestinal surgery ward and stoma nursing outpatient clinic of the study hospital; the primary researcher and two uniformly trained nursing staff personally approached potential participants. To ensure voluntary participation, eligible patients were fully informed that participation was optional and they could withdraw at any stage without negative impacts on clinical treatment or medical services. The research team provided detailed explanations of the study’s purpose, procedures, potential risks and benefits, responded to all patient questions, and obtained written informed consent before data collection; all collected data were anonymized (by removing personal identifiers) to protect privacy. Sample flow: A total of 310 patients were approached during the recruitment period, of which 300 were assessed as eligible based on inclusion/exclusion criteria, 20 eligible patients declined participation (mainly due to time constraints or lack of interest), and 280 eligible patients consented and were enrolled. All 280 enrolled participants received questionnaires (either paper-based or electronic, with the electronic version requiring full completion to avoid missing data and the paper version being reviewed on-site to supplement missing responses). Finally, 258 valid completed questionnaires were collected. Effective response rate calculation: The 95.6% effective response rate was calculated as (number of valid completed questionnaires / number of enrolled participants who received questionnaires) × 100%, which reflects the proportion of valid data obtained among the enrolled participants who received questionnaires. The combination of on-site verification for paper questionnaires, forced-response settings for electronic questionnaires, and thorough informed consent procedures ensured a high response rate and minimal data loss.

### 2.5 Statistical methods

Data were analyzed using SPSS version 26.0. The sample size for regression analyses was determined based on the widely accepted “5–10 events per variable” rule, a standard guideline to ensure model stability, reduce overfitting risk, and enhance result reliability. This study included 15 variables in regression analyses (8 sociodemographic variables, 1 self-efficacy variable, 1 social support variable, 1 eHL variable, 1 health-related quality of life variable, and 3 confounding variables: tumor location, disease stage, treatment regimen). Following the stricter “10 events per variable” criterion, the minimum required sample size was 150, and our 258 valid completed questionnaires far exceeded this requirement, ensuring sufficient statistical power. It should be specifically stated that the total scores of Likert-type scales (eHEALS, MSPSS, GSES) and the EQ-5D health utility index in this study were treated as continuous variables for analysis. This approach is methodologically justified based on two reasons: First, it is a common practice in the analysis of multi-item scale data. When a scale has a large number of items and a wide range of total scores, treating the total score as a continuous variable can fully utilize data information and enable the use of more efficient parametric statistical methods. Second, the sample size of this study is 258, which meets the criteria for a large sample. According to the Central Limit Theorem, even if the original data distribution is not completely normal, the sampling distribution of the mean tends to be normal, which provides sufficient support for the application of statistical methods based on continuous variables. Specific analysis of scale data: Descriptive statistics: Categorical variables from the General Information Questionnaire were described using frequencies and percentages; continuous variables (age, disease duration) and total scores of eHEALS, MSPSS, GSES, and HUI of EQ-5D-3L were described using means ± standard deviations (after confirming normality via the Shapiro–Wilk test). Inter-group comparisons: For continuous variables, independent samples *t*-tests were used for comparisons between two independent groups; one-way ANOVA (with Tukey *post-hoc* test) was used for comparisons among three or more independent groups. Linear trend tests were applied to ordered categorical variables to assess monotonic gradients in scale scores. Association exploration: Pearson correlation analysis was used to explore the linear relationship between two continuous variables. Multiple linear regression analysis (stepwise method) was used to explore the combined influence and independent contribution of multiple independent variables (sociodemographic variables, self-efficacy, social support) on a continuous dependent variable (eHEALS total score) while controlling for confounding factors (tumor location, disease stage, treatment regimen). Mediation analysis: The Baron and Kenny method was used to test whether eHEALS scores (mediating variable) mediated the relationship between predisposing/enabling factors (independent variables: self-efficacy, social support, sociodemographic variables) and EQ-5D-3L HUI (outcome variable). All statistical tests were two-tailed, with a significance level of *α* = 0.05 considered statistically significant.

## 3 Results

### 3.1 Descriptive statistics: eHEALS, MSPSS, GSES, and EQ-5D scores

A total of 258 individuals diagnosed with CRC were included in the analysis. The mean age of participants was 64.58 ± 12.07 years. The mean score on the eHEALS was 19.71 ± 8.97. Among the eHEALS subdomains, the highest mean score was recorded in the application of online health information and services, whereas the lowest was observed in the domain related to decision-making ability. The mean scores for the MSPSS, GSES, and EQ-5D were 63.4 ± 9.99, 23.39 ± 7.43, and 0.86 ± 0.16, respectively (Table 1).

**Table 1:** eHEALS, MSPSS, GSES, and EQ-5D scores among individuals with CRC (*n* = 258) (points, *X ± s*).

Items	Score
eHEALS	19.71 ± 8.97
Application of online health data and services	12.86 ± 5.85
Assessment ability	4.74 ± 2.44
Decision-making ability	2.12 ± 1.25
MSPSS	63.4 ± 9.99
GSES	23.39 ± 7.43
EQ-5D	0.86 ± 0.16

### 3.2 Comparison of eHEALS scores by participant characteristics

Univariate analysis demonstrated statistically significant differences in eHEALS scores based on marital status, educational attainment, income, living situation, and the number of concurrent chronic conditions (*p* < 0.05). These findings are detailed in Table 2.

**Table 2:** Comparison of eHEALS scores among individuals with CRC according to sociodemographic and clinical characteristics (*n* = 258).

Items	*n*	Percentage (%)	Score (points. X ± s)	*t* (F) value	*p* value
Sex				0.858	0.970
Male	172	66.7	20.05 ± 8.92		
Female	86	33.3	19.03 ± 9.08		
Age					
<60 years	91	35.3	26.97 ± 6.93	11.942	0.312
≥60 years	167	64.7	15.76 ± 7.34		
Marital status				6.153^a^	0.002
Married	240	93.0	19.96 ± 8.87		
Unmarried	2	0.8	34.5 ± 3.54		
Divorced/Widowed	16	6.2	14.13 ± 7.79		
Educational level				38.544^a^	<0.001
Primary school or below	62	24.0	13.13 ± 5.07		
Junior high school	126	48.8	19.02 ± 8.12		
High school/vocational high school	55	21.3	25.91 ± 8.42		
College education or above	15	5.8	30.07 ± 6.35		
Employment status				6.774	0.293
Employed	111	43.0	23.73 ± 7.95		
Unemployed	147	57.0	16.68 ± 8.52		
Type of medical insurance				5.346	0.415
Urban medical insurance	129	50.0	22.55 ± 8.75		
Rural cooperative medical insurance	129	50.0	16.88 ± 8.3		
Personal monthly income				9.540^a^	<0.001
Under 2,000 yuan	70	27.1	16.39 ± 8.95		
2,000–9,000 yuan	168	65.1	20.46 ± 8.71		
More than 9,000 yuan	20	7.8	25.05 ± 7.38		
Place of residence				4.355	0.240
Urban	138	53.5	21.91 ± 8.91		
Rural	120	46.5	17.19 ± 8.39		
Living situation				3.940^a^	0.009
Living alone	10	3.9	15.4 ± 10.43		
Living with spouse	103	39.9	21.86 ± 8.31		
Living with family	141	54.7	18.4 ± 9		
Living with friends	4	1.6	21.5 ± 11.62		
Self-assessed health status				1.482^a^	0.229
Poor	24	9.3	22.67 ± 8.81		
Fair	168	65.1	19.3 ± 8.89		
Good	66	25.6	19.68 ± 9.16		
Diagnosis				0.001^a^	0.999
Rectal cancer	182	70.5	19.7 ± 8.91		
Colon cancer	68	26.4	19.75 ± 8.84		
Rectosigmoid junction cancer	8	3.1	19.75 ± 12.34		
Disease course				1.439^a^	0.232
≤3 months	98	38.0	20.9 ± 9.05		
4 ~ 6 months	58	22.5	19.19 ± 8.71		
7 months~1 year	24	9.3	16.92 ± 7.84		
≥1 year	78	30.2	19.47 ± 9.3		
Pathological stage				1.005^a^	0.391
Stage I	59	22.9	20.92 ± 8.64		
Stage II	63	24.4	18.46 ± 8.46		
Stage III	81	31.4	19.23 ± 9.6		
Stage IV	55	21.3	20.56 ± 8.91		
Treatment regimen				−3.732	0.612
Surgery	192	74.4	18.52 ± 8.77		
Surgery + radiotherapy/chemotherapy	66	25.6	23.18 ± 8.7		
Presence of stoma				−1.193	0.551
Yes	206	79.8	19.38 ± 9.04		
No	52	20.2	21.04 ± 8.64		
Concurrent chronic conditions				8.075^a^	<0.001
No	96	37.2	21.84 ± 8.98		
One	89	34.5	18.12 ± 8.59		
Two or more	73	28.3	16.6 ± 8.27		

a Represents *F* value.

### 3.3 Correlation analysis of eHEALS with MSPSS, GSES, and EQ-5D

Pearson correlation analysis demonstrated significant positive correlations between that eHEALS scores and scores on the MSPSS, GSES, and EQ-5D (*p* < 0.01) (Table 3).

**Table 3:** Pearson correlation analysis of between eHEALS scores and MSPSS, GSES, and EQ-5D scores among individuals with CRC (*r* value).

Items	eHEALS	MSPSS	GSES	EQ-5D
eHEALS	1.000	—	—	—
MSPSS	0.360	1.000	—	—
GSES	0.741	0.496	1.000	—
EQ-5D	0.224	0.202	0.216	1.000

All *p* values < 0.01.

### 3.4 A associated factors of eHL in the multivariable analysis

A multiple linear stepwise regression analysis was performed with eHEALS scores as the dependent variable. Independent variables included MSPSS, GSES, and EQ-5D scores, as well as the sociodemographic and clinical variables identified as significant in the univariate analysis. The assignment of independent variables is presented in Supplementary Table 1. No multicollinearity was identified, as indicated by variance inflation factor (*VIF*) < 5.000 (*VIF*_min_ = 1.019, *VIF*_max_ = 1.796). The final model explained 65.8% of the total variance. Educational attainment, unmarried status, cohabitation with friends, and the presence of two or more concurrent chronic conditions were identified as significant factors influencing eHL (*p* < 0.01). After adjusting for potential confounding variables, including education, marital status, income, living situation, and comorbidity status, MSPSS was no longer a significant predictor (*p* > 0.05). In contrast, both GSES and EQ-5D scores were positively associated with eHL scores (*p* < 0.05), as presented in Table 4.

**Table 4:** Multiple linear stepwise regression analysis of factors influencing eHL among individuals with CRC.

Variable	*B*	*SE*	*Beta*	*t*	*p*	*VIF*
Constant	−3.812	1.971	—	−1.934	0.054	—
Unmarried	9.924	4.302	0.097	2.307	0.022	1.292
Junior high school	2.494	0.859	0.139	2.903	0.004	1.674
High school/vocational high school	6.468	1.086	0.296	5.954	<0.001	1.796
College education or above	7.811	1.716	0.204	4.553	<0.001	1.463
Living with friends	−8.289	2.887	−0.114	−2.871	0.004	1.154
Two concurrent chronic conditions	−3.825	0.883	−0.162	−4.331	<0.001	1.019
GSES	0.727	0.051	0.603	14.167	<0.001	1.317
EQ-5D	4.886	2.179	0.086	2.243	0.026	1.067

*R*^2^ = 0.658, △*R*^2^ = 0.647, *F* value = 59.780, *p* value < 0.001.

## 4 Discussion

We found that eHL was relatively low in this CRC cohort and was positively associated with self-efficacy and HRQoL after adjustment for sociodemographic factors. The mean eHEALS score among 258 patients with CRC in this study was 19.71 ± 8.97. This result is markedly lower than mean values reported in studies from other countries across diverse cultural and healthcare settings, indicating a substantial need for eHL improvement in this Chinese patient population (23). A cross-sectional study on American CRC survivors (*n* = 326, post-treatment follow-up cohort) found a mean eHEALS score of 28.6 ± 6.2 (24); a Canadian observational study on newly diagnosed CRC patients (*n* = 298) yielded a mean score of 27.3 ± 7.1 (25). Individuals with limited eHL are likely to struggle with effectively finding, understanding, and evaluating online health information. The observed disparity underscores that eHL is not a universal construct but is profoundly shaped by local socio-demographic and technological factors, thereby justifying the necessity for context-specific assessments and interventions in China. The lower score may be attributed to factors such as older, lower educational attainment, limited access to digital resources, reduced comprehension of health-related information, and challenges navigating or effectively using digital health technologies (26). Further analysis across eHL dimensions revealed that patients scored highest on items related to the application of online health information and services, indicating a relatively strong capacity for accessing health information. This is closely associated with the high penetration of smartphones in China and a general shift among patients toward using online platforms for health-related information. The availability of diverse digital content, such as health-related articles and education materials, coupled with the proliferation of mobile internet and short-video platforms, has diversified the formats of available information and significantly lowered the barrier to acquiring health knowledge (27). However, this convenience may lead individuals, particularly patients, to rely on superficial information without adequately evaluating its quality and credibility. Conversely, the decision-making ability dimension received the lowest score, primarily due to the vast amount yet variable quality of online health information (28). Information overload, compounded by patients’ limited ability to discern information, not only increases their cognitive burden and interferes with judgment but also makes it difficult to evaluate the authenticity, scientific validity, and applicability of information, thereby undermining rational decision-making (29).

Regarding influencing factors of eHL, this study identified several key variables with varying impacts among CRC patients. Multiple sociodemographic factors were independently associated with better eHL scores, including being married, having a higher education level, higher income, and fewer chronic comorbidities. Specifically, married patients likely benefit from spousal emotional support and daily care, which facilitates health information acquisition and utilization (29). In contrast, divorced or widowed patients often lack such support, potentially leading to loneliness or anxiety and diminished capacity for health information management. A positive gradient was observed between education level and eHEALS scores, with scores increasing progressively from “primary or below” to “college or above”, suggesting that higher education broadens information access and critical appraisal skills-underscoring the need for tailored support among less-educated patients (30, 31). Although personal monthly income showed a positive correlation with eHL in univariate analysis, it did not enter the final regression model, suggesting its effect might be mediated or confounded by other socioeconomic variables like education or occupational status (30). Furthermore, living situations and comorbidity number also influenced eHL. Patients living alone scored higher than those cohabiting with friends, possibly due to the latter’s lower socioeconomic status and limited health-related dialogue, which may promote information-avoidance (32). However, this subgroup was small, requiring cautious interpretation. Finally, a higher number of chronic comorbidities correlated with lower eHL, likely because multimorbidity increases cognitive burden, depletes energy, and provokes negative affect, thereby constraining health information management (33).

Notably, both general self-efficacy and EQ-5D scores emerged as independent positive predictors of eHL. General self-efficacy demonstrated the strongest explanatory power, as reflected by the highest standardized regression coefficient, a finding consistent with prior studies and Social Cognitive Theory (34, 35). Patients with high self-efficacy are more proactive in seeking, evaluating, and applying digital health information; they exhibit greater confidence in disease self-management, use health apps and online consultations more frequently, and possess an enhanced ability to overcome technical and informational barriers. In contrast, patients with low self-efficacy often avoid digital health tools due to apprehensions about their complexity or the unreliability of the content. The EQ-5D score, which reflects health-related quality of life (HRQoL), was also a key predictor of eHL. Better HRQoL enhances the physical and mental capacity to engage with health information, thereby facilitating activities such as online searches or appointment scheduling. For instance, patients with minimal post-operative pain or functional impairment are better positioned to perform these tasks efficiently. This observation aligns with previous findings that individuals with higher HRQoL demonstrate greater adaptability in health behaviors and are more likely to use online health services (36). These two factors jointly influence eHL through an interactive “capacity–condition” mechanism: self-efficacy, as a core “capacity” element, drives patients not only to acquire health knowledge but also to critically evaluate and apply health information; meanwhile, HRQoL, as a fundamental “condition” factor, provides the necessary foundation for the sustained and effective use of these digital tools.

The associations between eHL and four key factors are highly congruent with Andersen’s Behavioral Model and corroborated by international research while reflecting Chinese contextual characteristics: education and marital status lay the foundation of eHL, general self-efficacy bridges predisposing factors and enabling resources by reducing digital anxiety and motivating proactive engagement with electronic health information, and EQ-5D-measured HRQoL determines the capacity for eHL engagement by providing physical and mental well-being support—collectively forming a globally consistent yet locally adapted interactive framework; additionally, this study verified that eHL partially mediates the relationships between self-efficacy/educational level and HRQoL, confirming its role as a core enabling resource linking predisposing factors to positive HRQoL outcomes in line with Andersen’s model and international evidence. Based on these findings, targeted eHL interventions for Chinese CRC patients should adopt a multi-stakeholder collaborative approach grounded in Andersen’s model, including capacity building, environment optimization, and individualized care.

Based on the above findings, targeted intervention strategies to enhance the eHL of CRC patients can be developed from three main perspectives: First, healthcare professionals should prioritize providing tailored digital navigation support to patients with lower educational attainment or multiple comorbidities. It is advisable to assign dedicated digital navigators to these higher-risk groups, who can offer one-on-one guidance on using mainstream health information applications, interpreting electronic medical records, and identifying authoritative health information sources. Concurrently, the development and management of science communication platforms that utilize visual materials such as images and videos can help lower comprehension barriers and enhance the efficiency of improve information acquisition. Second, governments and healthcare institutions need to strengthen “Information Regulation and Environment Optimization” by standardizing the entire process of electronic health information collection, review, and dissemination, cracking down on false, exaggerated, or misleading information, and reducing the interference of information overload on patient decision-making. Third, clinical interventions should emphasize “Individual Differences and Multidisciplinary Collaboration.” This includes designing simplified and intuitive health materials for patients with lower education levels, establishing peer support groups and psychological support hotlines for divorced or widowed patients, enhancing self-efficacy through skill training. Furthermore, embedding brief, structured self-efficacy enhancement interventions or coaching sessions into scheduled CRC follow-up visits is recommended. For example, during post-operative follow-ups at 3, 6, and 12 months, healthcare providers could conduct standardized 15–20 min sessions focused on collaborative goal-setting, positive reinforcement of progress in using digital health tools, and problem-solving support for accessing reliable health information online. Additionally, there is a clear need to implement nurse-led digital literacy training programs specifically designed for older CRC patients with low EQ-5D scores. Given that study data indicate particular challenges among older patients with impaired quality of life, such training should adopt a modular format. Suggested content covers basic digital device operation, safe access to accredited health websites, and accurate interpretation of electronic medical reports. A proposed framework includes 4 to 6 sessions of 30 min each, with a follow-up evaluation 1 month after training to assess outcomes and adjust the intervention as needed. Ultimately, a multi-stakeholder collaborative approach is essential for systematically improving patients’ eHL levels.

This study has several limitations that should be considered when interpreting the results. First, the use of convenience sampling from a single Class A tertiary hospital in Taizhou, Jiangsu Province limits the generalizability of the findings. Patients recruited from this setting are likely to have better access to digital health resources, higher health literacy, and more favorable socioeconomic conditions compared to CRC patients in underdeveloped regions or primary/secondary medical institutions in China. Consequently, the relationships among predisposing factors, enabling resources (eHL), and need factors (health-related quality of life) observed in this study may not be applicable to the broader Chinese CRC patient population, particularly those with limited medical resources and digital access. Second, the cross-sectional design of the study prevents the establishment of causal relationships between variables; Future studies should adopt multi-center, prospective designs with random sampling covering both developed and underdeveloped regions to enhance the generalizability of results and explore causal relationships. Additionally, this study focused on eHL as a core enabling resource, but other potential enabling factors were not included, which may limit the comprehensiveness of the theoretical model’s application.

## 5 Conclusion

Overall, the level of eHL among individuals with CRC in China remains suboptimal, particularly in the domains related to evaluating and decision-making based on electronic health information. Significant variability in eHL was observed across participants with differing sociodemographic and clinical characteristics. Influencing factors included marital status, educational attainment, personal income, living situation, number of chronic comorbidities, self-efficacy, and health-related quality of life. In clinical settings, attention should be directed toward populations at elevated risk of low eHL. Enhancement strategies should be grounded in a dual framework of “ability improvement and environmental support.” Such strategies should aim to strengthen self-efficacy, improve health-related quality of life, promote active engagement in disease self-management, and ultimately contribute to better health outcomes.

## Data Availability

The raw data supporting the conclusions of this article will be made available by the authors, without undue reservation.
