# Supplementary material for: Associations of electronic health literacy with related factors in individuals diagnosed with colorectal cancer: a cross-sectional analysis
Source: Front Public Health. 2026 Feb 4;14:1681416. doi: 10.3389/fpubh.2026.1681416 (PMC12913524; doi:10.3389/fpubh.2026.1681416)
Supplement: Supplementary file 1 [file Table_1.docx]

Supplementary Table 1: Coding scheme for independent variables used in the multiple linear stepwise regression analysis influencing eHL

| Independent variable | Assignment method |
| --- | --- |
| Marital status | Married = 0, unmarried = 1, divorced/widowed = 2 |
| Educational level | Primary school or below = 0, junior high school = 1, high school/ vocational high school = 2, college education or above = 3 |
| Monthly personal income (RMB) | Less than 2,000 yuan = 0, 2,000-9,000 yuan = 1, more than 9,000 yuan = 2 |
| Living situation | Living alone = 0, living with spouse = 1, living with family = 2, living with friends = 3 |
| Concurrent chronic conditions | No = 0, one = 1, two or more = 2 |
| MSPSS | Enter original value |
| GSES | Enter original value |
| EQ-5D | Enter original value |
